# Human Aging Reflects Continuous Transcriptional Remodeling of the Donor Myocardium

**DOI:** 10.1096/fj.202603439R

**Published:** 2026-11-10

**Authors:** Cameron Chang, Pinki Dey, Ava E. Wraight, Hosen Kiat, Craig S. McLachlan, Amy Li

**Affiliations:** ^1^ Faculty of Medicine, Health and Human Sciences Macquarie University Sydney New South Wales Australia; ^2^ AXIS Research Health Institute Torrens University Australia Surry Hills New South Wales Australia; ^3^ Cardiac Health Institute Epping New South Wales Australia; ^4^ NICM Health Research Institute University of Western Sydney Sydney New South Wales Australia; ^5^ ANU School of Medicine and Psychology Australian National University Canberra Australian Capital Territory Australia; ^6^ School of Medical Sciences The University of Sydney Sydney New South Wales Australia; ^7^ Department of Rural Clinical Sciences, La Trobe Rural Health School La Trobe University Melbourne Victoria Australia

**Keywords:** adulthood, cardiac aging, donor heart, myocardial transcriptomics

## Abstract

Donor age in heart transplantation is constantly adapting. In this study we examine the molecular basis of cardiac aging and its relevance to donor heart assessment by analyzing transcriptomic data from left ventricular tissue of brain‐dead donor hearts not used for transplantation. Donors were stratified into young, middle‐aged, and older adult groups to characterize age‐associated transcriptional variation. The findings identified a continuous gradient of age‐associated gene expression without evidence of discrete molecular state transitions, and these changes were associated with coordinated remodeling of extracellular matrix, metabolic, immune, and neurohumoral pathways. Notably, 18% of the top 50 age‐associated transcripts were comprised of non‐coding RNAs. Older donor hearts exhibited overlapping molecular signatures with middle‐aged hearts rather than with a distinct transcriptomic profile. These findings support a continuum model of myocardial aging in donor hearts and confirm that older donor hearts should be evaluated beyond age, within a broader clinical and functional context.

## Graphical abstract

In this study, transcriptomic analysis of human donor myocardium revealed continuous age‐associated molecular changes across adulthood, without discrete age‐defined molecular states. Older donor hearts showed graded and overlapping transcriptional signatures with middle‐aged hearts, reflecting progressive remodeling of myocardial gene expression rather than threshold changes. These findings support considering donor age as a continuous biological variable and evaluating older donor hearts within a broader clinical and functional context to inform transplantation assessment.

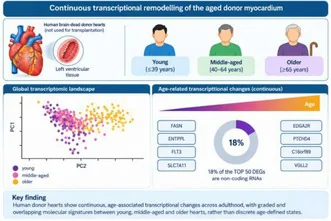

Donor hearts remain a limited pool with increasing age being a criterion for transplantation. As such defining the molecular signatures of cardiac aging has become increasingly relevant to heart transplantation [1]. Gene expression profiling can identify early molecular changes that precede overt functional decline and contribute to cardiovascular risk [1, 2]. Experimental animal models suggest transcriptional aging is gradual and linear [3]. Although emerging human studies demonstrate that aging trajectories vary between organs within the same individual [4] and may follow non‐linear patterns, with major molecular transitions occurring in the mid‐40s and early‐60s [5]. As these analyses were based on circulating markers rather than tissue‐specific transcriptomes, the molecular landscape of the human donor myocardium remains incompletely characterized. Human donor hearts provide a unique opportunity to study these processes in clinically relevant tissue. Although hearts from brain‐dead donors are subject to catecholamine surges, inflammatory activation and haemodynamic instability [6], these responses are inherent to transplant practice and reflect the physiological stressors of organ procurement.

Chronological age remains an important consideration for transplantation but is an imperfect surrogate for myocardial suitability. A key unresolved question is whether donor hearts exhibit discrete transcriptomic states that support age‐based exclusion preceding clinical procurement. To address this, we performed transcriptomic profiling of myocardial tissue from brain‐dead donor hearts procured but not used for transplantation to determine whether human donor myocardium exhibits discrete age‐associated molecular transitions. Donors were stratified into young (≤ 39 years, *n* = 18), middle‐aged (40–64 years, *n* = 98), and older (≥ 65 years, *n* = 45) groups to capture potential transitional biology across adulthood. The young group reflects early adulthood with low prevalence of overt cardiovascular disease and modifiable risk factors [7] while older individuals represent a stage beyond the second molecular transition [8] and are associated with increased cardiovascular risk.

Transcriptome dataset obtained from donor left ventricles was used for differential gene expression (DE) analysis performed in R (v4.3.0) using DESeq2 (v1.38.0). Genes with fewer than 20 counts in at least three samples were excluded. Significance was defined as FDR < 0.05 and |log_2_fold‐change| > 0.5. Variance‐stabilizing transformation was applied for downstream visualization. Chronological age was modeled as a continuous variable using linear and natural cubic spline regression (3 degrees of freedom). Fitted relationships and 95% confidence intervals were visualized to assess linear and non‐linear age‐associated expression patterns. Principal component (PC) analysis was performed on the transcriptomic profiles of individual donors annotated according to clinical characteristics. Association between each clinical characteristic and PC1/PC2, were quantified using Spearman's ρ for continuous phenotypes and *η* ^2^ for categorical phenotypes. These effect‐size measures are phenotype‐specific and are not directly comparable in magnitude. Spearman's ρ describes the direction and strength of the monotonic association (−1 to +1), whereas *η* ^2^ represents the proportion of PC variance attributable to differences between categorical groups. Gene set enrichment analysis was performed using clusterProfiler (v4.6.0), ranking genes by signed log_2_(fold‐change). KEGG pathway enrichment was assessed using a weighted Kolmogorov–Smirnov test, with pathways considered significant at FDR *q* < 0.05. All statistical analyses were conducted in R, and visualizations were generated using ggplot2 and pheatmap.

The human donor myocardium showed continuous transcriptional expression across adulthood. Heatmap analysis of the top differentially expressed genes revealed an age‐associated molecular gradient from young to older hearts with no abrupt transcriptional transitions (Figure 1A). Principal component analysis (PCA) was performed to assess the potential influence of clinical characteristics on sample clustering. PCA revealed no distinct clustering of samples across available clinical characteristics including age, sex, BMI, race, diabetes, hypertension, ventricular tachycardia/fibrillation, and atrial fibrillation (Figure 1B). These factors are unlikely to account for major sources of global transcriptional variation across the dataset. Our findings indicate that myocardial aging does not resolve into distinct molecular states. Broadly, expression levels were consistent across samples within each age group, although greater variability was observed in middle‐aged hearts, which may be indicative of asynchronous molecular remodeling between individuals [9].

**FIGURE 1:**
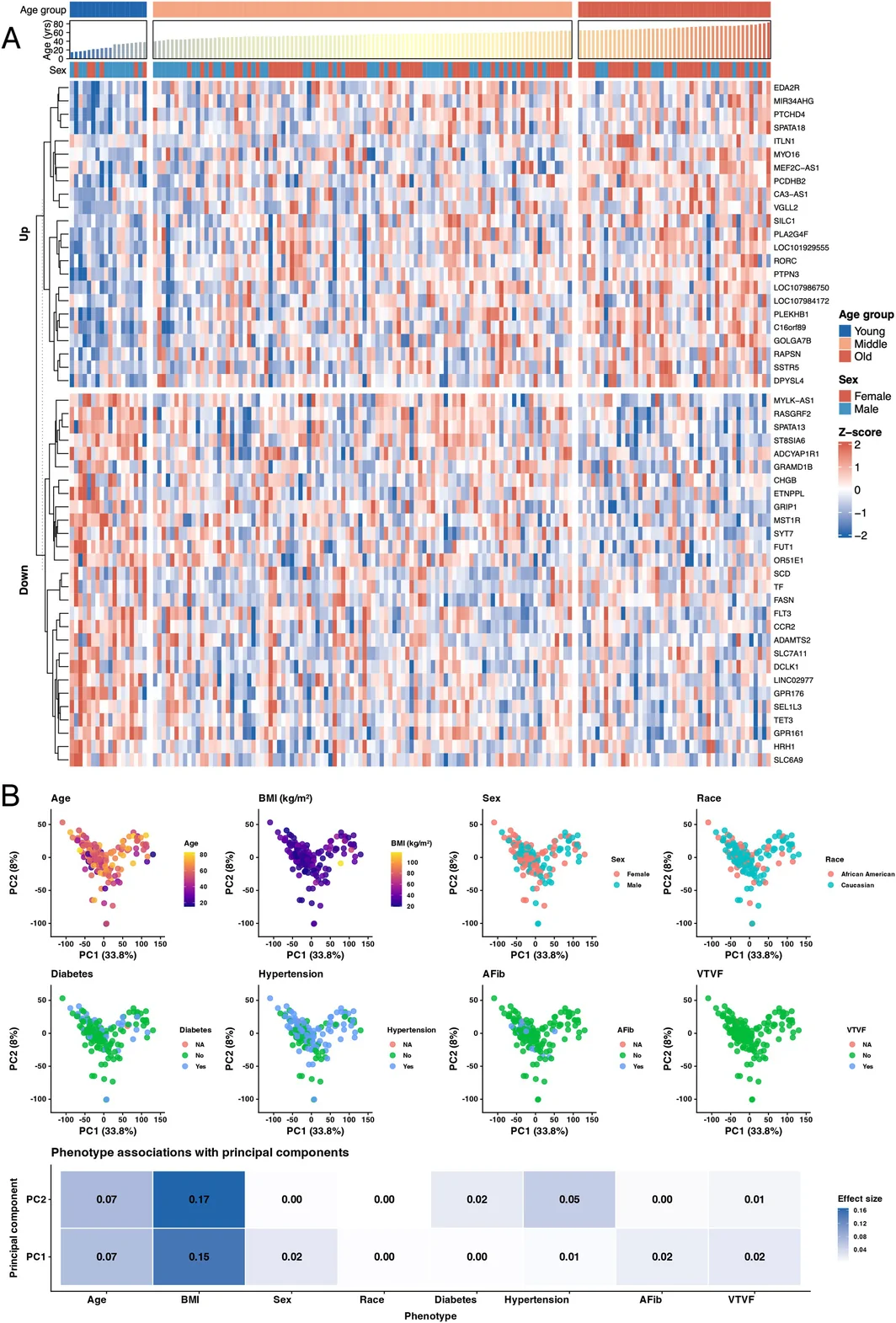
Age‐associated transcriptional expression profiles in left ventricular human donor myocardium. Samples are classified by age into young adults (≤ 39 years; *n* = 18), middle age (40–64 years; *n* = 98) and old age (≥ 65 years; *n* = 45). (A) Heatmap of the top 51 differentially expressed genes across donor hearts ordered by age (young to old). (B) PCA shows the distribution of individual donors annotated according to continuous (age and body mass index; BMI) or categorical (sex, race, diabetes, hypertension, atrial fibrillation; AFib, and ventricular fibrillation; VTVF) clinical characteristics. The transcriptomic variance accounted for by PC1 and PC2 is indicated on their respective axes. The accompanying phenotype–PCA heatmap quantifies the strength of association between each phenotype and the first two principal components.

Continuous age‐associated modeling of individual genes showed consistent directional expression profiles. Transcripts including EDA2R, PTCHD4, C16orf89, and VGLL2 demonstrated increasing expression with age, whereas FASN, FLT3, ENTPPL, and SLC7A11 expression was inversely associated with age (Figure 2A). While effect sizes varied between young, middle‐aged, and older comparisons, directionality was preserved, suggesting graded modulation of expression levels (Figure 2A). Notably, transcriptional remodeling with age extends beyond protein‐coding genes, with 18% of age‐associated transcripts comprised of non‐coding RNAs, such as MIR34AHG, MEF2C–AS1, CA3–AS1, LOC101929555, LOC107986750, LOC107984172, C16orf89, MYLK–AS1, and LINC02977 (Figure 1A).

**FIGURE 2:**
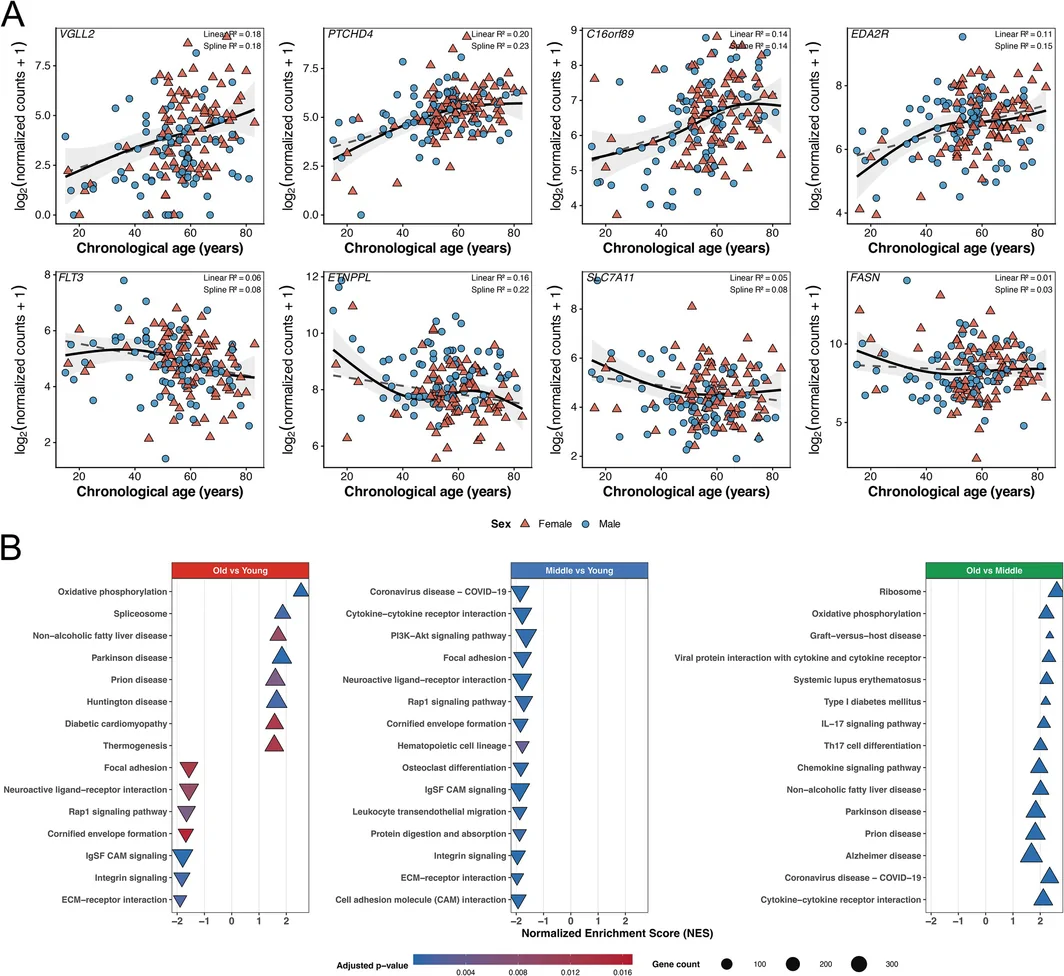
(A) Continuous modeling of age‐associated genes in non‐failing donor hearts. Eight differentially expressed genes were assessed using chronological age as a continuous variable, adjusted for sex. Selected genes show individual donor expression with linear (dashed) and spline (solid) age trajectories. Shaded areas indicate 95% confidence intervals. Linear and spline *R* ^2^ represent the proportion of expression variance explained by the respective sex‐adjusted models. (B) KEGG pathway enrichment analysis of differentially expressed genes across pairwise age group comparisons. Significance was defined as FDR < 0.05.

Among these transcripts, MIR34AHG suppresses cellular regeneration and proliferation [10, 11], and EDA2R promotes inflammatory signaling via NF‐κB [12]. These genes were recognized in aging and ischaemic injury, where their suppression reduces cell death and fibrosis to improve myocardial repair. Importantly EDA2R expression and C16orf89 expression were associated with increased atherosclerosis risk in healthy patients [13]. Within the present dataset, these genes represent an enrichment of immune and stress‐related transcriptional programs that becomes activated with advanced aging. Their increased expression may indicate reduced myocardial resilience to acute stress or injury, highlighting a period when donor hearts begin to show subtle molecular changes that could influence functional reserve, even in the absence of overt disease.

KEGG pathway analysis identified recurrent enrichment of extracellular matrix organization in young hearts, while metabolic regulation, immune, and neurohumoral signaling pathways were notable in older hearts (Figure 2B). Importantly, these pathways were present across multiple comparisons, indicating that age‐associated transcriptional variation reflects coordinated modulation of gene expression. Myocardial aging appears to reflect continuous and progressive adaptation across structural, metabolic, and signaling systems as opposed to discrete molecular transitions. These observations contrast with broader aging literature reporting heterogeneous and, in some cases, non‐linear molecular trajectories across tissues and circulating biomarkers [3, 4, 5]. In contrast to multi‐organ and plasma‐derived studies, the myocardial transcriptome does not show discrete biological thresholds with age but instead suggests that cardiac aging follows a continuous and integrated pattern of gene programming.

Clinically, these findings support the interpretation of donor age as a continuous biological variable rather than a categorical determinant of myocardial suitability. The absence of discrete transcriptional clustering indicates that older donor hearts do not form a separate age‐defined molecular state but instead demonstrate graded and overlapping molecular signatures with middle‐aged donors. The unequal distribution of donors across age groups, particularly the sampling of the young adult group, limits statistical power for comparisons involving this group and is an important consideration when interpreting age‐associated differences.

Donor hearts are also subject to physiological stressors associated with brain death and potential intensive care management, including catecholaminergic surges, inflammatory activation, and metabolic perturbation [6] which may contribute to the inter‐individual transcriptional variation. While this study provides mechanistic insight into donor heart biology, transcriptomic profiling alone cannot predict cardiac function, transplant suitability, post‐transplant outcomes, establish age cut‐offs, or serve as a standalone biomarker of myocardial aging. Integration of transcriptomic profiles with physiological measures, clinical parameters, and transplant outcomes may help resolve whether molecular signatures can contribute to more incremental assessment of aged donor myocardial assessment strategies.

Human donor myocardium exhibits continuous age‐associated transcriptional variation without evidence of discrete molecular state transitions. This reflects coordinated remodeling of myocardial gene expression across adulthood rather than age‐dependent threshold changes. These findings support a continuum‐based model of cardiac aging in donor hearts and further confirm that clinical evaluation beyond chronological age should be considered for transplantation procurement to expand the donor pool.

## Author Contributions

C.S.M. conceptualized the project, provided the resources, and supervision. A.L. conceptualized and provided project administration, formal analysis, and supervision. P.D. performed data curation and formal analysis. C.C. performed formal analysis, and writing – original draft preparation. A.E.W. participated in draft manuscript refinement. H.K. provided resources and clinical input. All authors were involved in writing – review and editing. All authors take responsibility for all aspects of the reliability and freedom from bias of the data presented and their discussed interpretation.

## Funding

The authors have nothing to report.

## Ethics Statement

The authors have nothing to report.

## Consent

The authors have nothing to report.

## Conflicts of Interest

The authors declare no conflicts of interest.

## Data Availability

Datasets used in this study were acquired from Gene Expression Omnibus; GSE141910. Analysis code is available upon reasonable request.
